# Macroscopic Tissue Integrity and Histological Diagnostic Yield During Endoscopic Ultrasound-Guided Tissue Acquisition for Solid Pancreatic Lesions

**DOI:** 10.3390/jcm15218281

**Published:** 2026-10-24

**Authors:** Ahmet Bektaş, Ömer Küçükdemirci, Bilge Can Meydan, Hakan Demiröz, Niyazi Görkem Bektaş, Filiz Karagöz, Mehmet Farsak, Ufuk Avcıoğlu

**Affiliations:** 1 Division of Gastroenterology, Department of Internal Medicine, Ondokuz Mayıs University School of Medicine, 55100 Samsun, Turkey; hakandemiroz.90@gmail.com (H.D.); mehmetfarsak@gmail.com (M.F.); ufukavcioglu@yahoo.com (U.A.); 2 Department of Medical Pathology, Ondokuz Mayıs University School of Medicine, Kurupelit-Atakum, 55100 Samsun, Turkey; canbilge@yahoo.com (B.C.M.); filizkaragoz@yahoo.com (F.K.); 3 Department of Anesthesiology, Akdeniz University Faculty of Medicine, 07070 Antalya, Turkey; bktsgorkem@gmail.com

**Keywords:** endoscopic ultrasound, EUS-guided tissue acquisition, solid pancreatic lesions, macroscopic on-site evaluation, cord sign, diagnostic yield

## Abstract

**Background and Aim:** Macroscopic on-site evaluation (MOSE) is being increasingly used during endoscopic ultrasound (EUS)-guided tissue acquisition when rapid on-site evaluation (ROSE) is unavailable. However, current MOSE criteria are primarily based on the presence or length of visible tissue fragments. The potential diagnostic significance of a macroscopically intact tissue specimen with preserved gross integrity has not been adequately investigated. This study evaluated the relationship between a macroscopically intact cord-like biopsy specimen (cord sign) and pathological diagnostic outcomes in solid pancreatic lesions. **Methods:** This retrospective cohort study included 380 patients who underwent EUS-guided biopsy for solid pancreatic lesions between January 2021 and December 2025. Cord sign positivity was defined as retrieval of a single intact tissue fragment measuring at least 1 cm with preserved macroscopic integrity and was assessed independently in formalin-fixed block and smear specimens. The primary outcome was histological diagnostic yield. Secondary outcomes included specimen adequacy, histological malignant or suspicious diagnosis, distribution of histological diagnoses according to overall cord sign status, and overall diagnostic success. Multivariable logistic regression analyses were performed to evaluate factors associated with histological diagnostic yield and, in an additional exploratory analysis, histological malignant or suspicious diagnosis. **Results:** A total of 380 patients were included in the final analysis, including 338 block cord sign-positive and 42 block cord sign-negative cases. Histological diagnostic yield was numerically higher in the block cord sign-positive group (82.2% vs. 71.4%, *p* = 0.098), although the difference was not statistically significant. Histological malignant or suspicious diagnosis was significantly more frequent among block cord sign-positive specimens (54.7% vs. 28.6%, *p* = 0.002), whereas specimen adequacy and combined diagnostic success were comparable between the groups. In the primary multivariable analysis, block cord sign positivity was not independently associated with histological diagnostic yield (OR 1.63, 95% CI 0.75–3.55, *p* = 0.218). However, an additional exploratory multivariable analysis demonstrated an association between block cord sign positivity and malignant or suspicious histological diagnosis (OR 3.44, 95% CI 1.56–7.59, *p* = 0.002). **Conclusions:** Block cord sign positivity was associated with a numerically higher histological diagnostic yield in patients undergoing endoscopic ultrasound-guided tissue acquisition for solid pancreatic lesions, although this association did not reach statistical significance in either the unadjusted or adjusted analyses. In contrast, malignant or suspicious histological findings were more frequently observed among block cord sign-positive specimens, and this association remained significant in an additional exploratory multivariable analysis. However, the potential influence of underlying lesion characteristics and the absence of an independently established final diagnosis prevent these findings from being interpreted as evidence that cord sign independently reflects specimen quality or predicts malignancy. Cord sign represents a practical macroscopic observation based on specimen length and preserved tissue integrity rather than a previously validated assessment criterion. Although its evaluation in both formalin-fixed block and smear specimens may provide a useful framework for further investigation, the present findings do not establish its role in guiding additional needle passes or improving diagnostic performance. Prospective multicenter studies using standardized and reproducible criteria, including formal interobserver agreement assessment, are needed to clarify its clinical relevance.

## 1. Introduction

Solid pancreatic lesions represent a heterogeneous group of diseases ranging from inflammatory masses to highly aggressive malignancies [1,2]. Among these, pancreatic ductal adenocarcinoma remains one of the most lethal cancers and is associated with a particularly poor prognosis [3]. Furthermore, with the increasing adoption of neoadjuvant treatment strategies in recent years, histopathological confirmation prior to treatment has become increasingly important. Owing to its high diagnostic accuracy and favorable safety profile, endoscopic ultrasound (EUS)-guided tissue sampling has become one of the standard approaches for the diagnosis of solid pancreatic lesions [4,5].

Rapid on-site evaluation (ROSE) has traditionally been used during EUS-guided sampling to assess specimen adequacy at the time of the procedure. However, because ROSE requires the availability of an experienced cytopathologist, its routine use remains limited in many centers [6,7]. For this reason, macroscopic on-site evaluation (MOSE), based on the visual appearance of the retrieved specimen, has become increasingly popular in daily practice. Several studies have shown that MOSE can provide satisfactory diagnostic outcomes and may represent a practical alternative when ROSE is not available [8].

In routine practice, endosonographers frequently notice that specimens considered adequate by MOSE can have markedly different macroscopic appearances [8]. Some samples consist of multiple small tissue fragments, whereas others are retrieved as a single, elongated tissue core with preserved gross integrity [9,10]. Although this observation is familiar to most operators performing EUS-guided biopsies, it has received little attention in the literature. Whether obtaining a macroscopically intact tissue core is associated with improved pathological diagnostic performance remains unknown.

On the other hand, EUS operators frequently encounter specimens that are retrieved as a single, grossly intact tissue core with preserved macroscopic integrity. Despite being a common observation in daily practice, this finding has not been clearly defined in the literature, and data regarding its potential diagnostic significance remain limited. Therefore, the aim of the present study was to evaluate the relationship between macroscopically intact, cord-like EUS biopsy specimens and pathological diagnostic success in patients with solid pancreatic lesions.

## 2. Materials and Methods

### 2.1. Study Design and Patient Selection

This single-center retrospective observational cohort study was conducted at the Department of Gastroenterology, Ondokuz Mayıs University, and was reported in accordance with the Strengthening the Reporting of Observational Studies in Epidemiology (STROBE) statement. The study protocol was approved by the Ondokuz Mayıs University Clinical Research Ethics Committee (Approval No. 2025/652; 25 December 2025).

All consecutive adult patients who underwent EUS-guided pancreatic tissue acquisition for a solid pancreatic lesion between January 2021 and December 2025 were retrospectively identified from the institutional endoscopy database and electronic medical records. To avoid within-patient correlation, only the first EUS-guided tissue acquisition procedure performed for each patient during the study period was included in the analysis.

Patients were excluded if they were younger than 18 years of age; had previously undergone EUS-guided pancreatic tissue acquisition; had a history of upper gastrointestinal surgery resulting in altered anatomy that precluded EUS examination; had received prior abdominal radiotherapy or systemic chemotherapy; had incomplete clinical, endoscopic, or pathological data; or had not provided consent for the use of their anonymized clinical data for research purposes. Patients who underwent EUS-guided tissue acquisition using dedicated fine-needle biopsy (FNB) needles were excluded, as the present study specifically focused on specimens obtained using conventional FNA needles.

### 2.2. EUS-Guided Tissue Acquisition and Dual-Specimen Processing

EUS examinations and tissue acquisition procedures were performed using a linear-array echoendoscope (EG-580UT; Fujifilm Medical Systems, Tokyo, Japan). All EUS-guided procedures were performed under anesthesiologist-directed intravenous deep sedation according to institutional routine practice.

All tissue acquisition procedures were performed by experienced endosonographers, each of whom had performed more than 1000 EUS examinations before the study period. Tissue acquisition was performed using 22G or 25G Areus Premium EUS-FNA needles (Micro-Tech, Nanjing, China), featuring an elongated dual-edged bevel tip. Needle size was selected according to lesion location, technical accessibility, and the endosonographer’s discretion.

For all lesions, tissue acquisition was performed using the stylet slow-pull technique combined with the fanning technique. A single needle pass was defined as one needle insertion into the target lesion, during which approximately 10 to-and-fro movements were performed under continuous EUS guidance. Lesion location, maximum lesion diameter, needle gauge, and the total number of passes were prospectively documented in the institutional endoscopy reporting system as part of routine clinical practice.

Because rapid on-site cytological evaluation (ROSE) was not available, macroscopic assessment of all retrieved specimens was routinely performed immediately after tissue acquisition.

During routine specimen processing, the material obtained from the initial needle pass was first expelled onto a glass slide using the stylet for macroscopic assessment. When sufficient material was observed on the slide, the remaining material within the needle was subsequently expelled into 10% neutral buffered formalin for histopathological processing. When necessary, the material on the initial glass slide was distributed across additional slides to prepare conventional cytological smears, which were immediately fixed in 95% ethanol. Conventional cytological smears were prepared from the retrieved material regardless of whether a macroscopically intact cord-like tissue fragment was identified. The formalin-fixed material was assessed macroscopically, and specimens containing a single intact cord-like tissue fragment measuring at least 1 cm were considered cord sign positive. Accordingly, both cytological and histological specimens could be obtained during a single needle pass in some patients. When the retrieved material was considered insufficient, additional needle passes were performed at the discretion of the endosonographer according to the macroscopic appearance of the specimen and routine clinical judgment. The formalin-fixed specimens were processed for histopathological examination according to the standard protocol of the Department of Pathology.

### 2.3. Cord Sign Assessment

Macroscopic assessment of retrieved specimens was conceptually similar to macroscopic on-site evaluation (MOSE) described in the literature. However, unlike conventional MOSE criteria, which primarily focus on the amount or visible length of tissue obtained, our assessment also considered preservation of macroscopic tissue integrity.

For the purposes of this study, specimens retrieved as a single intact cord-like tissue fragment measuring at least 1 cm with preserved gross tissue integrity, irrespective of tissue color, were considered cord sign positive. Specimens composed of multiple small tissue fragments or lacking a grossly intact appearance were considered cord sign negative. Specifically, smear cord-negative status was defined as the absence of a single intact cord-like tissue fragment measuring at least 1 cm on the glass-slide specimen, including preparations containing fragmented tissue or dispersed cellular material. In cases where the initially retrieved specimen was considered insufficient for cord sign assessment, additional sampling was performed at the discretion of the endosonographer. If an intact cord-like specimen was obtained during subsequent passes, the specimen was recorded as cord sign positive. Specimen length was assessed macroscopically using a ruler available at the specimen-handling station. The volume graduations on the formalin containers provided additional approximate visual reference points but were not used as calibrated length measurements.

Cord sign was assessed independently in both formalin-fixed block specimens and glass-slide specimens by the operating endosonographer together with at least one experienced gastroenterologist or senior gastroenterology fellow trained in advanced endoscopy, and the findings were recorded as part of the routine procedural documentation. Accordingly, block cord sign and smear cord sign were analyzed as independent variables (Figure 1). For additional analyses, overall cord positivity was defined as positivity in either smear or block specimens, whereas overall cord negativity required absence of cord sign in both preparations.

This standardized dual-specimen processing protocol enabled independent assessment of cord sign in formalin-fixed and glass-slide specimens while providing separate material for histopathological and cytological evaluation.

### 2.4. Pathological Evaluation and Study Outcomes

All histopathological specimens were evaluated by pathologists experienced in gastrointestinal pathology who were blinded to cord sign status. For the purposes of this study, histological diagnostic yield was defined as the proportion of specimens providing an interpretable histopathological diagnostic category, including benign, malignant, atypia, suspicious for malignancy/neoplasia, and dysplasia/other neoplasm. Specimens reported as fibrin, blood clot, necrotic material, inadequate tissue, or otherwise lacking an interpretable histopathological finding were classified as non-diagnostic. This classification was refined during manuscript revision to include atypia and suspicious for malignancy/neoplasia as interpretable histopathological findings since these categories indicate the presence of diagnostically relevant cellular or tissue material despite not establishing a definitive diagnosis. Accordingly, histological diagnostic yield was distinguished from definitive diagnostic accuracy, while specimens containing only non-informative material remained classified as non-diagnostic.

Cytological specimens were categorized as malignant, suspicious for malignancy, atypical, benign, or non-diagnostic according to the Papanicolaou Society of Cytopathology terminology [11,12].

The primary study outcome was histological diagnostic yield. Secondary outcomes included block adequacy, smear adequacy, histological malignant or suspicious diagnosis, distribution of histological diagnoses according to overall cord sign status, overall diagnostic success, and factors independently associated with histological diagnostic yield. For the secondary outcome of histological malignant or suspicious diagnosis, only specimens categorized as adenocarcinoma/carcinoma/metastasis or suspicious for malignancy/neoplasia were considered positive.

### 2.5. Statistical Analysis

Statistical analyses were performed using IBM SPSS Statistics for Windows Version 25.0 (IBM Corp., Armonk, NY, USA). Continuous variables are expressed as mean ± standard deviation or median (minimum–maximum), as appropriate. Categorical variables are presented as frequencies and percentages. Comparisons between cord sign-positive and cord sign-negative groups were performed using Student’s *t*-test or Mann–Whitney U test according to data distribution. Categorical variables were compared using the Chi-square test or Fisher’s exact test when appropriate. Logistic regression analysis was performed to evaluate factors associated with histological diagnostic yield. An additional exploratory multivariable logistic regression analysis was performed to investigate factors associated with histological malignant or suspicious diagnosis. Both regression models included age, sex, lesion size, needle caliber, total number of passes, and block cord sign status as independent variables. Multivariable analyses were performed using a complete-case approach, excluding patients with missing data for any variable included in the models. No imputation of missing values was performed. Results are expressed as odds ratios (ORs) with 95% confidence intervals (CIs). A *p*-value < 0.05 was considered statistically significant.

## 3. Results

A total of 380 EUS-guided tissue acquisition procedures for solid pancreatic lesions were included in the final analysis. Of these, 338 were block cord sign-positive and 42 were block cord sign-negative.

Patients with a positive block cord sign were significantly older than those without a cord sign (64.3 ± 11.9 vs. 58.4 ± 16.7 years, *p* = 0.033). No significant differences were observed between the groups regarding sex distribution, lesion size, lesion location, needle caliber, or total number of passes (Table 1).

Histological diagnostic yield was higher among patients with a positive block cord sign compared with those without a cord sign, although the difference did not reach statistical significance (82.2% vs. 71.4%, *p* = 0.098). Similarly, the rate of histological malignant or suspicious diagnoses was significantly higher in the cord sign-positive group (54.7% vs. 28.6%, *p* = 0.002). Combined diagnostic success, defined as obtaining a diagnostic result from either histology or cytology, was comparable between the groups (93.8% vs. 92.9%, *p* = 0.739). No significant differences were observed in specimen adequacy rates. Block adequacy was comparable between the two groups (86.7% vs. 85.4%, *p* = 0.809), as was smear adequacy (86.1% vs. 81.0%, *p* = 0.357) (Table 2).

When patients were categorized according to smear and block cord sign patterns, histological diagnostic yield differed numerically among the four groups, although the overall comparison did not reach statistical significance (*p* = 0.087). The highest diagnostic yield was observed in the smear-negative/block-positive group (89.8%), followed by the smear-positive/block-negative (81.8%) and smear-positive/block-positive (80.6%) groups, whereas the lowest yield was observed in specimens negative for cord sign in both smear and block preparations (67.7%). In an exploratory pairwise comparison, the smear-negative/block-positive group demonstrated a higher histological diagnostic yield than the double-negative reference group (89.8% vs. 67.7%, *p* = 0.018). However, the overall four-group comparison was not statistically significant (*p* = 0.087), and this finding should therefore be interpreted cautiously. The overall cord-positive group also showed a numerically higher diagnostic yield (82.2% vs. 67.7%, *p* = 0.057) (Table 3).

Histological diagnoses were further analyzed according to overall cord sign status (Table 4, Figure 2). Adenocarcinoma/carcinoma/metastatic lesions were significantly more frequent among patients with overall cord positivity (49.6% vs. 25.8%, *p* = 0.014). Conversely, benign/reactive lesions were more common in patients without cord sign (12.9% vs. 3.7%, *p* = 0.041). No statistically significant differences were observed for neuroendocrine tumors, dysplasia, suspicious lesions, atypia, or non-diagnostic specimens, although non-diagnostic specimens tended to be more frequent in the overall cord-negative group (32.3% vs. 17.8%, *p* = 0.057).

Multivariable logistic regression analysis was performed to identify independent predictors of histological diagnostic yield. Of the 380 patients included in the primary analyses, 358 had complete data for all variables included in the regression model and were therefore included in the multivariable analysis. After adjustment for age, sex, lesion size, needle caliber, and total number of passes, block cord sign positivity was associated with numerically higher odds of obtaining a diagnostic histological specimen, although this association did not reach statistical significance (OR 1.63, 95% CI 0.75–3.55, *p* = 0.218). None of the other variables included in the model were independently associated with histological diagnostic yield. Among the 358 patients included in the primary multivariable regression analysis, 288 had diagnostic histological findings and 70 had non-diagnostic findings (Table 5).

An additional exploratory multivariable logistic regression analysis was performed in the same 358 patients to investigate factors associated with histological malignant or suspicious diagnosis. After adjustment for age, sex, lesion size, needle caliber, and total number of passes, block cord sign positivity was associated with higher odds of obtaining a malignant or suspicious histological diagnosis (OR 3.44, 95% CI 1.56–7.59, *p* = 0.002). Increasing age (OR 1.03 per year, 95% CI 1.01–1.05, *p* = 0.006), greater lesion size (OR 1.03 per mm, 95% CI 1.02–1.05, *p* < 0.001), and a higher number of passes (OR 1.44, 95% CI 1.08–1.91, *p* = 0.012) were also independently associated with malignant or suspicious histological diagnosis. Among the same 358 patients included in the exploratory multivariable regression analysis, 185 had malignant or suspicious histological findings, whereas 173 had other histological outcomes (Table 6).

## 4. Discussion

In this cohort of patients with solid pancreatic lesions undergoing EUS-guided tissue acquisition, block cord sign positivity was associated with a higher observed histological diagnostic yield, although this difference did not reach statistical significance and was not independently significant in the adjusted analysis. In contrast, malignant or suspicious histological findings were significantly more frequent among block cord sign-positive specimens, and this association remained statistically significant in an additional exploratory multivariable analysis. Notably, neither block nor smear adequacy differed significantly between the groups, and combined diagnostic success was also comparable. These observations suggest that the macroscopic characteristics captured by the cord sign definition, including specimen length and preserved tissue integrity, may be related to certain histopathological outcomes, although their independent relationship with overall histological diagnostic yield and the potential influence of underlying lesion characteristics remain uncertain. While macroscopic evaluation of EUS-guided tissue acquisition has been investigated previously within the framework of MOSE, most studies have focused on the presence or length of visible core tissue [13,14,15]. In contrast, the potential relationship between preservation of gross tissue integrity and diagnostic performance has received relatively little attention.

The concept of macroscopic assessment during EUS-guided tissue acquisition is not new. Since the original MOSE study by Iwashita et al., several investigators have demonstrated that gross inspection of retrieved tissue material can provide useful information regarding specimen adequacy and diagnostic performance [13,16]. Most MOSE studies have defined adequacy according to the presence of a visible whitish core or the length of the retrieved tissue fragment, commonly using thresholds ranging from 4 mm to 10 mm. Subsequent studies and meta-analyses have reported diagnostic accuracies exceeding 90% when MOSE is used during EUS-guided tissue acquisition and have suggested that MOSE may represent a practical alternative in settings where ROSE is unavailable [10,13]. Most previous MOSE studies have focused on the presence of visible core tissue or the length of the retrieved specimen [16,17]. In daily practice, however, specimens that meet these criteria may have markedly different appearances. While some consist of multiple small tissue fragments, others are retrieved as a single continuous tissue core. In the present study, we specifically evaluated this latter appearance. Although histological diagnostic yield was numerically higher in cord sign-positive specimens, the difference was not statistically significant, and the independent contributions of specimen length and preserved macroscopic integrity could not be determined.

One of the notable findings of this study was that specimen adequacy and diagnostic performance did not necessarily coincide. Although block and smear adequacy rates were similar between cord sign-positive and cord sign-negative specimens, histological diagnostic yield was numerically higher in the cord sign-positive group without reaching statistical significance, whereas malignant or suspicious histological diagnoses were significantly more frequent. Combined diagnostic success was also comparable between the groups. These results indicate that obtaining material considered adequate for pathological evaluation does not always translate into an interpretable histopathological diagnosis.

This observation also highlights an important difference between our study and the existing MOSE literature. Most MOSE studies have assessed specimen adequacy according to the presence of a visible whitish core or the length of the retrieved tissue fragment, with visible tissue fragments measuring approximately 4 mm or more generally regarded as adequate [10,18]. Consequently, specimens composed of multiple tissue fragments may still fulfill conventional MOSE criteria [19]. In our study, however, cord sign positivity required retrieval of a single tissue fragment with preserved macroscopic continuity, irrespective of tissue color. Therefore, specimens containing tissue fragments that would otherwise be considered adequate by conventional MOSE standards were classified as cord sign negative if tissue integrity was not preserved. Despite this, cord sign-positive specimens showed a numerically higher histological diagnostic yield, although the difference did not reach statistical significance. Similarly, the adjusted analysis demonstrated a positive direction of association, but block cord sign was not independently associated with histological diagnostic yield at the conventional threshold for statistical significance. Moreover, lesion size, pass number, and needle caliber were not independently associated with diagnostic yield in the multivariable model. These findings suggest that the relationship between cord sign positivity and histological diagnostic yield remains uncertain and may be influenced by factors not fully accounted for in the present analysis. Taken together, the observed trend warrants further investigation, although the relative contributions of specimen length and preserved tissue integrity cannot be determined from the present data.

Much of the current literature on specimen assessment has evolved alongside advances in EUS-guided tissue acquisition, including the increasing use of EUS-FNB. Several meta-analyses have evaluated differences in diagnostic performance between FNA and FNB, particularly in the absence of ROSE [20]. In parallel, MOSE has become a commonly used approach for macroscopic assessment of retrieved specimens when ROSE is unavailable [19,21]. Contemporary pancreatic cancer management increasingly requires tissue not only for establishing a diagnosis but also for histological characterization and ancillary molecular analyses [22,23]. Although dedicated FNB needles are increasingly preferred for histological tissue acquisition, conventional FNA needles remain an important option in routine clinical practice, particularly in settings where cost considerations and limited access to advanced sampling devices influence procedural choices. In our institution, conventional FNA needles are more readily available and relatively less expensive than dedicated FNB needles, and ROSE is not routinely available. Against this background, we specifically focused on FNA-based tissue acquisition to investigate whether the retrieval of a macroscopically intact, elongated tissue fragment could provide potentially useful information regarding histopathological outcomes, even without dedicated FNB needles or on-site cytopathological evaluation. The association between cord sign and histological diagnostic performance therefore represents an assessment of a macroscopic specimen characteristic rather than a comparison between tissue acquisition techniques. Although our findings do not establish that cord sign improves diagnostic yield during FNA, they suggest that macroscopic assessment of tissue integrity may warrant further investigation as a practical approach in resource-limited clinical settings. Whether the diagnostic relevance of cord sign differs according to needle platform or acquisition technique warrants further investigation.

An additional finding emerged when smear and block cord sign status were evaluated together. Although histological diagnostic yield varied numerically among the four cord sign patterns, the overall comparison did not reach statistical significance. The highest diagnostic yield was observed in the smear-negative/block-positive group, which demonstrated a higher yield than the double-negative reference group in an exploratory pairwise comparison. However, this finding should be interpreted cautiously because the overall four-group comparison was not statistically significant and subgroup sizes were relatively small. Nevertheless, this observation suggests that preservation of tissue integrity within the formalin-fixed block specimen may have a closer relationship with histological diagnostic success than the macroscopic appearance of material prepared for cytological examination. This finding is biologically plausible. Histological diagnosis relies largely on preservation of tissue architecture and cellular relationships, whereas smear preparations are primarily intended for cytological evaluation [24,25,26,27]. A cord-like specimen retrieved into the formalin container may therefore retain architectural information that can be partially lost during smear preparation. While the present study was not designed to compare block and smear processing techniques directly, the observed pattern may indicate that cord sign assessment in block material deserves further investigation in future studies. Importantly, cord sign has not been validated as a decision rule for additional needle passes, and its absence alone should not be considered an indication for further sampling.

An additional methodological aspect of the present study is that cord sign was assessed independently in both formalin-fixed block specimens and glass-slide specimens rather than relying on a single preparation. In our unit, tissue obtained during EUS-guided sampling is routinely separated into histological and cytological preparations as part of standard clinical practice, allowing independent macroscopic assessment of both specimens. Importantly, both preparations could be obtained during a single needle pass when sufficient material was retrieved, without necessarily requiring separate needle insertions. Rather than introducing an additional procedural step, this approach reflects routine daily practice while enabling separate evaluation of tissue integrity in two independently processed preparations. Whether independent assessment of macroscopic tissue integrity in both preparations provides additional procedural information beyond conventional MOSE warrants further investigation [13,15].

Histological diagnoses were further evaluated according to overall cord sign status. Adenocarcinoma/carcinoma/metastatic lesions were significantly more frequent among specimens demonstrating overall cord positivity, whereas benign/reactive lesions were relatively more common in the absence of cord sign. This difference in histological distribution raises the possibility that underlying lesion characteristics may influence the likelihood of obtaining a macroscopically intact tissue fragment. Nevertheless, cord positivity was observed across almost all histological categories, including benign and non-diagnostic specimens. Therefore, cord sign should not be interpreted as a marker of malignancy itself. In the additional exploratory multivariable analysis, block cord sign positivity was associated with higher odds of obtaining a malignant or suspicious histological diagnosis (OR 3.44, 95% CI 1.56–7.59, *p* = 0.002). However, this association may partly reflect differences in the underlying lesion types, and the absence of an independently established final diagnosis prevents us from determining whether cord sign reflects specimen characteristics independently of lesion biology. Cytological and histological examination do not always reflect the same aspects of a pancreatic lesion [28,29]. As highlighted by Mizutani et al., discordance between cytological and histological diagnoses is not uncommon, and both methods may contribute complementary rather than interchangeable diagnostic information [30]. The high frequency of cord positivity observed among adenocarcinoma/carcinoma and neuroendocrine tumor cases is also in keeping with the recognized importance of preserved tissue architecture for histological interpretation and ancillary pathological analyses in these lesions [22,31,32]. At the same time, the presence of cord sign in benign and non-diagnostic specimens indicates that this macroscopic finding is not restricted to malignant lesions, although its relationship with tissue quality and underlying lesion characteristics requires further investigation.

Several limitations should be considered when interpreting the findings of this study. The retrospective single-center design may have introduced selection bias and limits the generalizability of the results. In addition, cord sign is not a previously validated classification system. Rather, it is a practical term that has long been used in our unit to describe biopsy specimens retrieved as a single grossly intact tissue fragment during EUS-guided sampling. Because this appearance was considered potentially relevant during macroscopic assessment of specimen quality, its presence or absence was routinely documented in procedural reports throughout the study period. Nevertheless, cord sign assessment was based on operator observation, and formal interobserver agreement was not evaluated. Although the defining feature of cord sign was retrieval of a single intact tissue fragment, specimen length was assessed macroscopically during the procedure using a ruler available at the specimen-handling station. The formalin containers also had volume graduations, with approximately 0.8 cm between adjacent markings, which provided additional visual reference points but were not used as calibrated length measurements. However, exact specimen lengths were not systematically recorded for individual patients, precluding a direct comparison of tissue length between cord sign-positive and cord sign-negative specimens. Furthermore, because cord sign positivity required both a minimum tissue length of approximately 1 cm and preservation of macroscopic integrity, the independent contributions of specimen length and tissue continuity to histological diagnostic yield could not be determined. As such, some degree of subjective variation in cord sign classification cannot be excluded. Although the total number of needle passes was routinely documented, the specific pass during which cord sign positivity was first achieved was not systematically recorded. Consequently, a pass-specific analysis or sensitivity analysis restricted to the initial needle pass could not be performed. Moreover, because additional sampling was performed at the discretion of the endosonographer, differences in lesion accessibility or sampling difficulty may have influenced the likelihood of cord sign positivity, introducing potential procedural selection bias. Furthermore, the primary outcome included interpretable histopathological categories such as atypia and suspicious for malignancy, which do not necessarily represent definitive clinical diagnoses. Therefore, histological diagnostic yield as defined in this study should not be interpreted as equivalent to definitive diagnostic accuracy. Another important limitation is the absence of an independently established final diagnosis for all lesions. Consequently, the observed association between cord sign positivity and malignant or suspicious histological findings may partly reflect differences in the underlying lesion types rather than an independent relationship with specimen quality. The exploratory multivariable analysis should therefore not be interpreted as evidence that cord sign independently predicts malignancy. Furthermore, the study exclusively included procedures performed using conventional FNA needles, without a comparative FNB group. Although this approach reflected our intention to investigate cord sign in a setting where conventional FNA was more readily accessible and ROSE was unavailable, the applicability of our findings to dedicated FNB needle platforms remains uncertain. No direct conclusions regarding the comparative diagnostic performance of FNA and FNB can be drawn from the present study. Finally, multivariable analyses were restricted to patients with complete data for all model variables, and missing values were not imputed. Although this approach ensured consistent model denominators, the possibility of bias related to incomplete records cannot be excluded. Some subgroup analyses also included a limited number of patients and should be interpreted cautiously. Prospective multicenter studies using predefined cord sign criteria, standardized sampling protocols, and independent validation are needed to further clarify the clinical relevance of this observation.

## 5. Conclusions

Block cord sign positivity was associated with a numerically higher histological diagnostic yield that did not reach statistical significance, and a significantly higher frequency of histological malignant or suspicious findings in patients undergoing EUS-guided tissue acquisition for solid pancreatic lesions. However, combined diagnostic success was comparable between the groups, and block cord sign positivity was not independently associated with histological diagnostic yield in the adjusted analysis. Given the retrospective design of the study and the relatively small number of patients in some subgroups, these findings should be interpreted with caution. Furthermore, cord sign does not represent a previously validated pathological or endoscopic descriptor. Rather, it reflects a macroscopic observation routinely documented in our clinical practice and evaluated in the present study because of its potential relationship with specimen characteristics. Although cord sign was observed across both benign and malignant lesions, the higher frequency of malignant or suspicious histological findings among cord-positive specimens may partly reflect differences in underlying lesion characteristics rather than specimen quality alone. In addition, independent assessment of cord sign in both formalin-fixed block and smear specimens may represent a practical methodological approach for future studies evaluating macroscopic specimen characteristics during EUS-guided tissue acquisition. Prospective multicenter studies using predefined and reproducible criteria are needed to further clarify the clinical relevance and potential clinical utility of this observation.

## Figures and Tables

**Figure 1:**
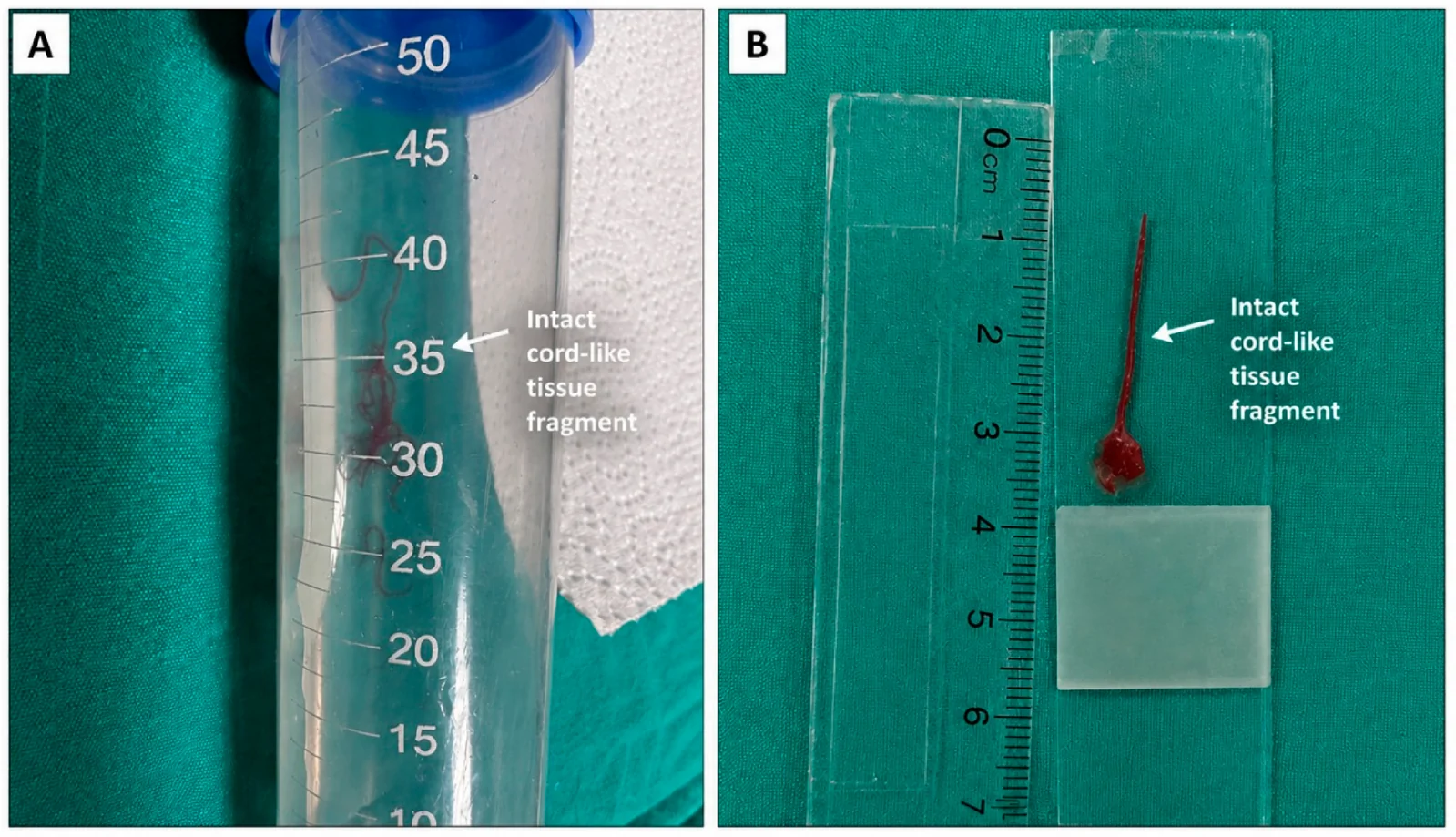
Representative examples of cord sign positivity. Hint: (**A**) Block cord sign positivity. The specimen was retrieved as a single intact cord-like tissue fragment immersed in formalin. (**B**) Smear cord sign positivity. The specimen was retrieved as a single intact cord-like tissue fragment before cytological preparation. In both examples, the cord length is ≥1 cm.

**Figure 2:**
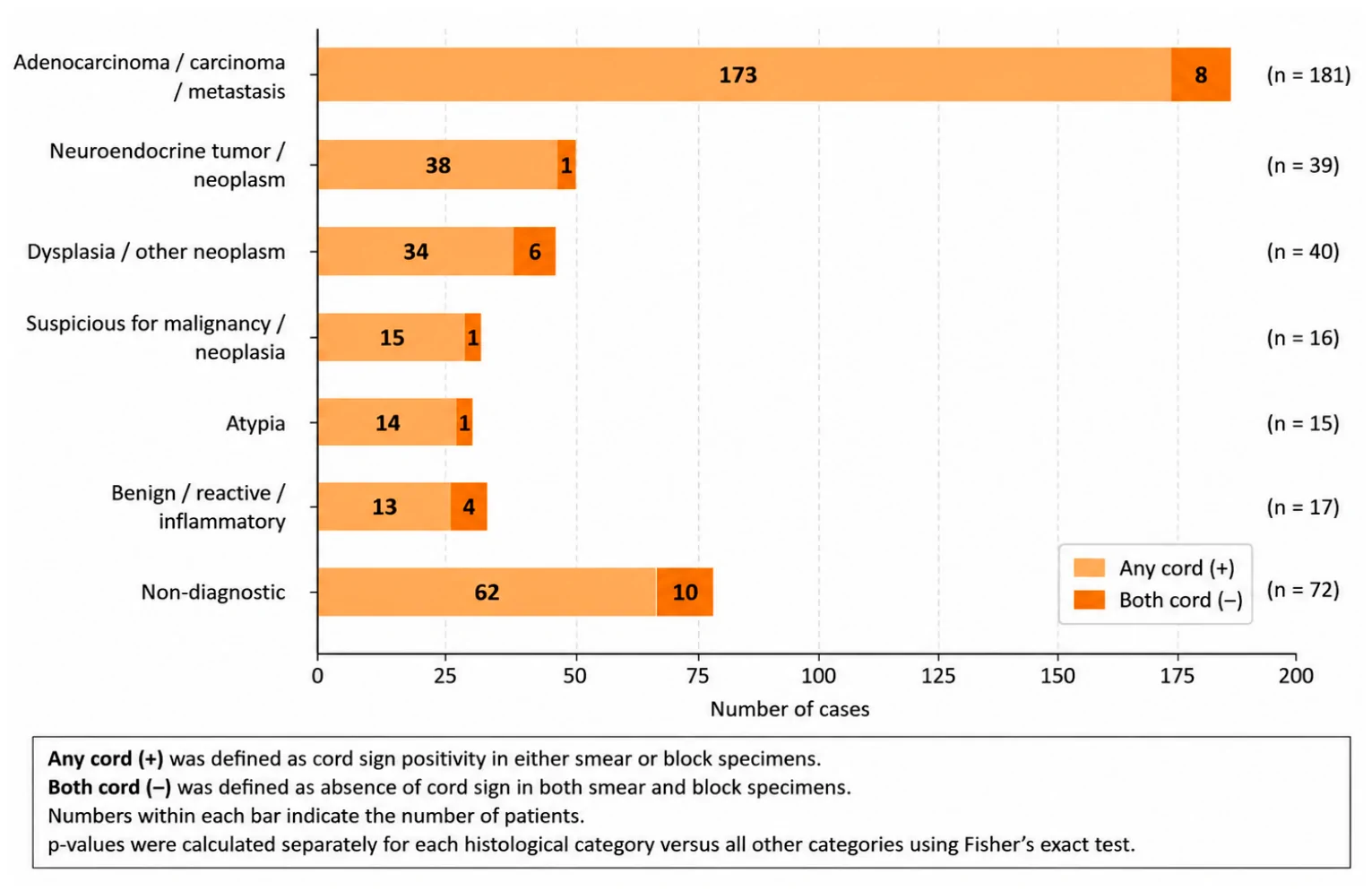
Distribution of Histological Diagnoses According to Overall Cord Sign Status.

**Table 1:** Baseline Demographic and Procedural Characteristics According to Block Cord Sign.

Variable	Cord Sign (−) *n* = 42	Cord Sign (+) *n* = 338	*p*-Value
Age, years	58.4 ± 16.7	64.3 ± 11.9	0.033
Male sex, n (%)	20 (47.6)	209 (61.8)	0.094
Lesion size, mm	32.0 ± 18.9	32.2 ± 14.4	0.948
Pancreatic head/uncinate location, n (%)	21 (50.0)	210 (62.1)	0.135
Pancreatic body location, n (%)	15 (35.7)	102 (30.2)	0.481
Pancreatic tail location, n (%)	6 (14.3)	24 (7.1)	0.124
22G needle, n/N (%)	32/41 (78.0)	259/332 (78.0)	1.000
25G needle, n/N (%)	9/41 (22.0)	73/332 (22.0)	1.000
Total number of passes	3.0 ± 1.0	2.7 ± 0.8	0.097

Abbreviations: G, gauge. Note: Percentages were calculated using available data for each variable; therefore, denominators may differ because of missing observations. Note: Two block cord sign-positive cases were recorded as involving the papilla of Vater and as a diffuse mass, respectively, and were therefore not assigned to the pancreatic head/uncinate, body, or tail categories.

**Table 2:** Diagnostic Outcomes According to Block Cord Sign.

Outcome	Cord Sign (−) n = 42	Cord Sign (+) n = 338	*p*-Value
Histological diagnostic yield	30/42 (71.4)	278/338 (82.2)	0.098
Block adequacy	35/41 (85.4)	293/338 (86.7)	0.809
Smear adequacy	34/42 (81.0)	291/338 (86.1)	0.357
Histological malignant/suspicious diagnosis	12/42 (28.6)	185/338 (54.7)	0.002
Combined diagnostic success	39/42 (92.9)	317/338 (93.8)	0.739

Histological malignant/suspicious diagnosis: Histological specimens reported as malignant or suspicious for malignancy. Combined diagnostic success: Achievement of a diagnostic result by either histological or cytological examination.

**Table 3:** Histological Diagnostic Yield According to Smear and Block Cord Sign Patterns.

Cord Pattern	n	Diagnostic Histology, n (%)	*p*-Value vs. Smear (−)/Block (−)
Smear (−)/Block (−)	31	21 (67.7)	Reference
Smear (+)/Block (−)	11	9 (81.8)	0.464
Smear (−)/Block (+)	59	53 (89.8)	0.018
Smear (+)/Block (+)	279	225 (80.6)	0.103
Any cord (+)	349	287 (82.2)	0.057

Overall four-group comparison: *p* = 0.087 (Pearson’s chi-square test). Note: Any cord (+) was defined as cord sign positivity in either smear or block specimens. *p*-values were calculated against the Smear (−)/Block (−) reference group using Fisher’s exact test. Pairwise subgroup comparisons were exploratory and were not adjusted for multiple testing.

**Table 4:** Distribution of Histological Diagnoses According to Overall Cord Sign Status.

Histological Diagnosis	Any Cord (+) n = 349	Both Cord (−) n = 31	*p*-Value
Adenocarcinoma/carcinoma/metastasis	173 (49.6)	8 (25.8)	0.014
Neuroendocrine tumor/neoplasm	38 (10.9)	1 (3.2)	0.230
Dysplasia/other neoplasm	34 (9.7)	6 (19.4)	0.119
Suspicious for malignancy/neoplasia	15 (4.3)	1 (3.2)	1.000
Atypia	14 (4.0)	1 (3.2)	1.000
Benign/reactive/inflammatory	13 (3.7)	4 (12.9)	0.041
Non-diagnostic	62 (17.8)	10 (32.3)	0.057

Note: Any cord (+) was defined as cord sign positivity in either smear or block specimens. Both cord (−) was defined as absence of cord sign in both smear and block specimens. *p*-values were calculated separately for each histological category versus all other categories using Fisher’s exact test.

**Table 5:** Multivariable Logistic Regression for Histological Diagnostic Yield.

Variable	OR	95% CI	*p*-Value
Block cord sign (+)	1.63	0.75–3.55	0.218
Age, per year	1.01	0.99–1.03	0.275
Male sex	0.80	0.46–1.39	0.419
Total passes	1.08	0.78–1.49	0.644
Lesion size, per mm	1.01	0.99–1.03	0.317
25G needle	0.72	0.39–1.34	0.302

Abbreviations: OR, odds ratio; CI, confidence interval. Variables entered into the multivariable model included age, sex, lesion size, needle caliber, total number of passes, and block cord sign status. The 22G needle was used as the reference category for needle gauge. Complete-case analysis was performed without imputation of missing data.

**Table 6:** Exploratory Multivariable Logistic Regression for Histological Malignant/Suspicious Diagnosis.

Variable	OR	95% CI	*p*-Value
Block cord sign (+)	3.44	1.56–7.59	0.002
Age, per year	1.03	1.01–1.05	0.006
Male sex	0.89	0.56–1.42	0.627
Total passes	1.44	1.08–1.91	0.012
Lesion size, per mm	1.03	1.02–1.05	<0.001
25G needle	0.67	0.39–1.15	0.148

Abbreviations: OR, odds ratio; CI, confidence interval. Variables entered into the multivariable model included age, sex, lesion size, needle caliber, total number of passes, and block cord sign status. The 22G needle was used as the reference category for needle gauge. This exploratory analysis was performed using a complete-case approach without imputation of missing data.

## Data Availability

The data supporting the findings of this study are not publicly available due to ethical and patient confidentiality considerations. However, anonymized data may be made available by the corresponding authors upon reasonable request, subject to applicable ethical approvals.

## References

[1] Dietrich C.F., Sahai A.V., D’Onofrio M., Will U., Arcidiacono P.G., Petrone M.C., Hocke M., Braden B., Burmester E., Möller K. (2016). Differential diagnosis of small solid pancreatic lesions. Gastrointest. Endosc..

[2] Chen J., Yang R., Lu Y., Xia Y., Zhou H. (2012). Diagnostic accuracy of endoscopic ultrasound-guided fine-needle aspiration for solid pancreatic lesion: A systematic review. J. Cancer Res. Clin. Oncol..

[3] Afghani E., Klein A.P. (2022). Pancreatic adenocarcinoma: Trends in epidemiology, risk factors, and outcomes. Hematol./Oncol. Clin..

[4] Gan Q., Roy-Chowdhuri S., Duose D.Y., Stewart J.M., Coronel E., Bhutani M.S., Lee J.H., Weston B., Ge P.S., Ross W.A. (2022). Adequacy evaluation and use of pancreatic adenocarcinoma specimens for next-generation sequencing acquired by endoscopic ultrasound–guided FNA and FNB. Cancer Cytopathol..

[5] Chung M.J., Park S.W., Kim S.-H., Cho C.M., Choi J.-H., Choi E.K., Lee T.H., Cho E., Lee J.K., Song T.J. (2021). Clinical and technical guideline for endoscopic ultrasound (EUS)-guided tissue acquisition of pancreatic solid tumor: Korean Society of Gastrointestinal Endoscopy (KSGE). Clin. Endosc..

[6] Lu Q., Dai T., Liu P., Shi R., He T., Zhang W. (2026). EUS-FNA with ROSE for pancreatic solid lesions: A single-center experience. BMC Gastroenterol..

[7] Gadour E., Miutescu B., Al Ghamdi S., Burciu C., Shaaban H., Vuletici D., Almuhaidb A., Ratiu I., Aljahdli E., Okasha H. (2025). Diagnostic accuracy and sensitivity of the rapid on-site evaluation (ROSE) versus macroscopic on-site evaluation (MOSE) in endoscopic ultrasound (EUS)-guided sampling: A systematic review. Frontline Gastroenterol..

[8] Ishikawa T., Ohno E., Mizutani Y., Iida T., Uetsuki K., Yashika J., Yamada K., Gibo N., Aoki T., Kataoka K. (2022). Usefulness of macroscopic on-site evaluation using a stereomicroscope during EUS-FNB for diagnosing solid pancreatic lesions. Can. J. Gastroenterol. Hepatol..

[9] Ogura T., Hijioka S., Hara K., Nishioka N., Okuda A., Ueno S., Nishikawa H., Yamada M., Nagashio Y., Hisada Y. (2024). Multicenter, randomized controlled trial of EUS-guided fine-needle biopsy using a fork-tip needle with macroscopic or rapid on-site evaluation for pancreatic lesions (H2O trial). Endosc. Ultrasound.

[10] Deng M., Luo D., Tan X., Zhou X., Zhao M., Yang P., Chen Y., Liang X., Sun X., Shan J. (2025). Application of Macroscopic On-Site Evaluation With Adjustable Light Source in EUS-FNA for Solid Pancreatic Masses. J. Clin. Gastroenterol..

[11] Pitman M.B., Centeno B.A., Ali S.Z., Genevay M., Stelow E., Mino-Kenudson M., Fernandez-del Castillo C., Max Schmidt C., Brugge W., Layfield L. (2014). Standardized terminology and nomenclature for pancreatobiliary cytology: The Papanicolaou Society of Cytopathology guidelines. Diagn. Cytopathol..

[12] Layfield L.J., Ehya H., Filie A.C., Hruban R.H., Jhala N., Joseph L., Vielh P., Pitman M.B. (2014). Utilization of ancillary studies in the cytologic diagnosis of biliary and pancreatic lesions: The Papanicolaou Society of Cytopathology guidelines for pancreatobiliary cytology. Diagn. Cytopathol..

[13] Iwashita T., Yasuda I., Mukai T., Doi S., Nakashima M., Uemura S., Mabuchi M., Shimizu M., Hatano Y., Hara A. (2015). Macroscopic on-site quality evaluation of biopsy specimens to improve the diagnostic accuracy during EUS-guided FNA using a 19-gauge needle for solid lesions: A single-center prospective pilot study (MOSE study). Gastrointest. Endosc..

[14] Mohan B.P., Madhu D., Reddy N., Chara B.S., Khan S.R., Garg G., Kassab L.L., Muthusamy A.K., Singh A., Chandan S. (2022). Diagnostic accuracy of EUS-guided fine-needle biopsy sampling by macroscopic on-site evaluation: A systematic review and meta-analysis. Gastrointest. Endosc..

[15] Sundaram S., Chhanchure U., Patil P., Seth V., Mahajan A., Bal M., Kaushal R.K., Ramadwar M., Prabhudesai N., Bhandare M. (2023). Rapid on-site evaluation (ROSE) versus macroscopic on-site evaluation (MOSE) for endoscopic ultrasound-guided sampling of solid pancreatic lesions: A paired comparative analysis using newer-generation fine needle biopsy needles. Ann. Gastroenterol..

[16] Guda N.M. (2026). Diagnosis of pancreatic cancers: Beyond image enhancement. Gastrointest. Endosc..

[17] Adler D.G., Chun M. (2025). ROSE Versus MOSE for Evaluation of EUS–Guided Tissue Samples. Pract. Gastroenterol..

[18] Chung K.H., Lee S.H. (2025). Optimal tissue acquisition method for pancreatic mass. Dig. Endosc..

[19] Chong C.C., Ligresti D., Kim T.H., Tarantino I., Traina M., Liotta R., Ip P.C., Lok H.T., Fung A.K., Kung J.W. (2026). Contrast-enhanced endoscopic ultrasound versus conventional endoscopic ultrasound–guided fine-needle biopsy with macroscopic on-site evaluation for solid pancreatic lesions: A multicenter randomized trial. Gastrointest. Endosc..

[20] Khan M.A., Grimm I.S., Ali B., Nollan R., Tombazzi C., Ismail M.K., Baron T.H. (2017). A meta-analysis of endoscopic ultrasound–fine-needle aspiration compared to endoscopic ultrasound–fine-needle biopsy: Diagnostic yield and the value of onsite cytopathological assessment. Endosc. Int. Open.

[21] Mangiavillano B., Frazzoni L., Togliani T., Fabbri C., Tarantino I., De Luca L., Staiano T., Binda C., Signoretti M., Eusebi L.H. (2021). Macroscopic on-site evaluation (MOSE) of specimens from solid lesions acquired during EUS-FNB: Multicenter study and comparison between needle gauges. Endosc. Int. Open.

[22] Kovacevic B., Toxværd A., Klausen P., Larsen M.H., Grützmeier S., Detlefsen S., Karstensen J.G., Brink L., Hassan H., Høgdall E. (2023). Tissue amount and diagnostic yield of a novel franseen EUS-FNB and a standard EUS-FNA needle—A randomized controlled study in solid pancreatic lesions. Endosc. Ultrasound.

[23] Bellocchi M.C.C., Bernuzzi M., Brillo A., Bernardoni L., Amodio A., De Pretis N., Frulloni L., Gabbrielli A., Crinò S.F. (2024). EUS-FNA versus EUS-FNB in pancreatic solid lesions≤ 15 mm. Diagnostics.

[24] Olyaee M., Jani B.S., Rzouq F., Saligram S., Lim D., Rastogi A., Bonino J. (2016). Endoscopic ultrasound-guided fine-needle aspiration of pancreatic lesions: A systematic review of technical and procedural variables. N. Am. J. Med. Sci..

[25] Hunt G.C., Smith P.P., Faigel D.O. (2003). Yield of tissue sampling for submucosal lesions evaluated by EUS. Gastrointest. Endosc..

[26] Wani S., Muthusamy V.R., Komanduri S. (2014). EUS-guided tissue acquisition: An evidence-based approach (with videos). Gastrointest. Endosc..

[27] Yousri M., Abusinna E., Tahoun N., Okasha H.H., El-Habashi A.H. (2022). A comparative study of the diagnostic utility of endoscopic ultrasound-guided fine needle aspiration cytology (EUS-FNA) versus endoscopic ultrasound-guided fine needle biopsy (EUS-FNB) in pancreatic and non-pancreatic lesions. Asian Pac. J. Cancer Prev. APJCP.

[28] Kopelman Y., Marmor S., Ashkenazi I., Fireman Z. (2011). Value of EUS-FNA cytological preparations compared with cell block sections in the diagnosis of pancreatic solid tumours. Cytopathology.

[29] Nigam N., Rastogi A., Bhatia V., Sureka B., Jain P., Bihari C. (2019). EUS-guided FNA in diagnosing pancreatic lesions: Strength and cytological spectrum. J. Cytol..

[30] Mizutani N., Mochizuki M., Toki M. (2020). Assessment of preoperative pancreatic biopsy, cytological/histological review of cell-block-specimens obtained by endoscopic ultrasound-guided fine-needle aspiration: Laboratory-based study. Diagn. Cytopathol..

[31] Tanaka H., Matsusaki S. (2022). The utility of endoscopic-ultrasonography-guided tissue acquisition for solid pancreatic lesions. Diagnostics.

[32] Cui M., Amed M., Reid M.D., Xue Y. (2025). Secondary pancreatic tumors in endoscopic ultrasound-guided fine-needle biopsy: Clinicopathologic characteristics and morphological diagnostic challenges. Hum. Pathol..

